# Revealing the genome of the microsporidian *Vairimorpha bombi*, a potential driver of bumble bee declines in North America

**DOI:** 10.1093/g3journal/jkae029

**Published:** 2024-02-09

**Authors:** Victoria L Webster, Samuel Hemmings, Marta Pérez, Matthew C Fisher, Mark J F Brown, Rhys A Farrer

**Affiliations:** Department of Biological Sciences, Royal Holloway University of London, London TW20 0EX, UK; MRC Centre for Global Infectious Disease Analysis, Imperial College London, London W2 1PG, UK; Department of Biological Sciences, Royal Holloway University of London, London TW20 0EX, UK; MRC Centre for Global Infectious Disease Analysis, Imperial College London, London W2 1PG, UK; Department of Biological Sciences, Royal Holloway University of London, London TW20 0EX, UK; MRC Centre for Medical Mycology, University of Exeter, Exeter EX4 4QD, UK

**Keywords:** *Vairimorpha bombi*, microsporidia, genome assembly, secretome, spore wall proteins, histone acetyltransferase

## Abstract

Pollinators are vital for food security and the maintenance of terrestrial ecosystems. Bumblebees are important pollinators across northern temperate, arctic, and alpine ecosystems, yet are in decline across the globe. *Vairimorpha bombi* is a parasite belonging to the fungal class Microsporidia that has been implicated in the rapid decline of bumblebees in North America, where it may be an emerging infectious disease. To investigate the evolutionary basis of pathogenicity of *V. bombi*, we sequenced and assembled its genome using Oxford Nanopore and Illumina technologies and performed phylogenetic and genomic evolutionary analyses. The genome assembly for *V. bombi* is 4.73 Mb, from which we predicted 1,870 protein-coding genes and 179 tRNA genes. The genome assembly has low repetitive content and low GC content. *V. bombi*'s genome assembly is the smallest of the *Vairimorpha* and closely related *Nosema* genera, but larger than those found in the *Encephalitozoon* and *Ordospora* sister clades. Orthology and phylogenetic analysis revealed 18 core conserved single-copy microsporidian genes including the histone acetyltransferase (HAT) *GCN5*. Surprisingly, *V. bombi* was unique to the microsporidia in not encoding the second predicted HAT *ESA1*. The *V. bombi* genome assembly annotation included 265 unique genes (i.e. not predicted in other microsporidia genome assemblies), 20% of which encode a secretion signal, which is a significant enrichment. Intriguingly, of the 36 microsporidian genomes we analyzed, 26 also had a significant enrichment of secreted signals encoded by unique genes, ranging from 6 to 71% of those predicted genes. These results suggest that microsporidia are under selection to generate and purge diverse and unique genes encoding secreted proteins, potentially contributing to or facilitating infection of their diverse hosts. Furthermore, *V. bombi* has 5/7 conserved spore wall proteins (SWPs) with its closest relative *V. ceranae* (that primarily infects honeybees), while also uniquely encoding four additional SWPs. This gene class is thought to be essential for infection, providing both environmental protection and recognition and uptake into the host cell. Together, our results show that SWPs and unique genes encoding a secretion signal are rapidly evolving in the microsporidia, suggesting that they underpin key pathobiological traits including host specificity and pathogenicity.

## Introduction

Microsporidia are globally ubiquitous and obligate pathogens that infect and subsequently impact the health of a wide range of animal species. Infection can be caused by both vertical and horizontal transmission, with the most common mode in humans (causing the disease Microsporidiosis) *via* zoonosis or ingestion of spores in food or water (Han, Takvorian, *et al.* 2020). Microsporidia infect their hosts by adherence of the spore wall proteins (SWPs) to the host cell surface prior to spore activation (Southern *et al.* 2007). Next, the spore is activated through polarization and changes to the spore wall, causing the polar tube to rapidly discharge out of the spore and pierce the host cell membrane, where it serves as a conduit for sporoplasm passage into the new host cell (Xu and Weiss 2005). The genomes of numerous species of Microsporidia have been sequenced, in order to understand both their evolution and their pathogenicity, revealing that they possess highly reduced genomes and consequently reduced numbers of protein-coding genes (Jespersen *et al.* 2022).

Microsporidia can have impacts beyond host health when they infect species that provide essential ecosystem services. Pollination, which is predominantly carried out by bees, is essential for crop production and ecosystem health (IPBES 2016). Honeybees and bumblebees, which provide the majority of pollination services, are infected by at least three species of Microsporidia—*Vairimorpha apis, V. bombi, and V. ceranae*—all of which impact host health (Grupe and Quandt 2020). The genomes of the predominantly honeybee-infecting microsporidia (*V. apis* and *V. ceranae*) have been sequenced (Chen *et al.* 2013; Huang *et al.* 2021), revealing important features of their genome evolution, including reduced genome length, reduced number of protein-coding genes, and loss of the RNAi protein Dicer. Indeed, Dicer has been reported to be lost in approximately half of all microsporidian species (Huang *et al.* 2021), where it otherwise would be involved in RNAi-driven silencing of transposons and viruses. *V. ceranae* has also been found in bumblebees, despite being primarily a honeybee-infecting parasite (Arbulo *et al.* 2015). Further comparative genomics work is needed to understand the evolutionary history and selective pressures that are acting on these *Vairimorpha* species, and this requires sequencing of the *V. bombi* genome.

*Vairimorpha bombi* (previously known as *Nosema bombi*; Tokarev *et al.* 2020) is an obligate fungal parasite of bumblebees in the Microsporidia family and has been endemic in European bumblebees for over a century (Fantham and Porter 1914). It significantly reduces colony fitness in its European bumblebee hosts (Otti and Schmid-Hempel 2007; Rutrecht and Brown 2009), and thus is likely to play an important role in bumblebee population dynamics. However, recently *V. bombi* has been proposed to be the cause of massive population declines in bumblebee species native to the United States (Whittington and Winston 2004). More specifically, it has been suggested that commercial bumblebee production facilities resulted in the spillover of European strains of *V. bombi* into North American bumblebees and that this spillover drove collapses in the populations of numerous native bumblebees (Thorp and Shepherd 2005). Analyses of temporal patterns of bumblebee decline and *V. bombi* prevalence provide support for this hypothesis (Cameron *et al.* 2011, 2016). However, the small subunit rRNA from *V. bombi* collected in Europe and the United States in 2010–2011 was genetically indistinct from North American museum samples collected from 1979 to 2011 (Cameron *et al.* 2016), leaving the origin of this putative spillover event unclear.

The genome of *V. bombi* has not yet been sequenced, but the development of a high-quality annotated assembly for *V. bombi* is essential to assess the risk it poses to global bumblebee populations. Such an assembly would facilitate a comparative genomics study exploring the pathogenicity and host specificity of bumblebee-infecting microsporidia, as well as whether a cross-continental spillover event is behind the collapse in North American bumblebee populations. Furthermore, an assembly is essential for future epidemiological investigations that may reveal mitigation strategies to limit the impact and continuing spread of *V. bombi*. Here, we have sequenced and assembled the *V. bombi* genome and used comparative genomics across the wider family of Microsporidia to predict the evolutionary origin of the species and the genetic causes of its pathogenicity and host specificity.

## Methods

### Spore purification

**Fig. 1.**
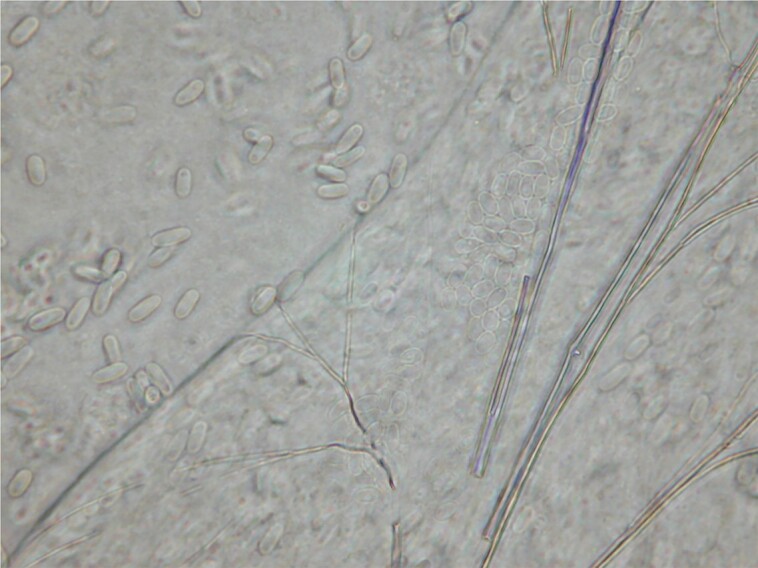
A photograph of a squash of Malpighian tubules from a bumblebee infected with *Vairimorpha bombi* at 400× magnification under phase contrast, showing spores both within and outside the tubules. © Mark J. F. Brown.

A wild *B. terrestris* queen that was naturally infected with *V. bombi* (strain VR1) was collected from Windsor Great Park, UK (SU992703) in 2016 (Fig. 1) (Folly *et al.* 2020) and used to inoculate eight *B. terrestris* colonies (Folly *et al.* 2021) which were then stored at −80°C until use. The abdomens from 200 of these infected workers were individually homogenized in 2 ml of 0.01 M NH_4_Cl to inhibit spore germination (Undeen and Avery 1988; Fries *et al.* 2013) and strained through layered cheesecloth to remove large particles. The spores were then isolated using density gradient centrifugation (10.11G for 10 minutes at 4°C, after which NH_4_Cl was removed and replaced, with this process being repeated until the pellet was pure white) and resuspended in sterile NH_4_Cl. Individual suspensions were pooled into two samples, each containing spores from 100 bees, and their final concentrations were quantified using a hemocytometer (Neubauer cell-counter chamber) under 400× magnification. Both suspensions were then pelleted again as before.

### DNA extraction and sequencing

To extract spore DNA for short-read sequencing, the pellet was suspended in yeast cell lysis solution (Biosearch technologies) with 1.0 mm Zirconia/Silica beads (Thistle Scientific) and disrupted in a TissueLyser II at 30 m/s for 45 seconds six times to mechanically break the spore walls. We then centrifuged the sample at 20.82G for two minutes, added 1 µl of RNase A, incubated at 65°C for 15 minutes, and then incubated on ice for 15 minutes. The DNA was then extracted and purified using the DNeasy Blood and Tissue Kit (Qiagen) and quantified using a Qubit dsDNA BR Assay (Qubit). We obtained 223 ng/μl in 38 μl from 1.29 × 10^7^ cells which we split into six tubes, each containing 25 ng/μl in 25 μl. Three tubes were sent to Novogene (Cambridge, UK) for sequencing using the Illumina HiSeq 4000 (San Diego, CA) using microbial whole genome library preparation (350 bp), and the remaining three were sent to the Earlham Institute (Norwich, UK) for sequencing using the Illumina NovaSeq 6000 (San Diego, CA) with reagent kit SP v1.5.

Long-read sequencing was performed on an Oxford Nanopore MinION flow cell. We minimized shearing of the DNA by encouraging the spores to germinate rather than breaking open the spore wall. We did this by desiccating the pellet of 4.37 × 10^7^ spores overnight at room temperature and then re-suspending them in 0.01 M Ringer's solution and incubating them for 3 hours at 30°C to simulate the environment inside the bee. We then extracted the DNA from this suspension using the DNeasy Plant Mini Kit (Qiagen). From this, we obtained 62 ng/μl of DNA in 18 μl which we prepared for sequencing using the library protocol SQK-LSK112. This DNA was loaded into a flow cell and sequenced on an Oxford Nanopore MinION flow cell (FLO-MIN106D; Oxford Nanopore Technologies) for 58 hours in total over the course of three runs, with no adaptive sampling. We set a Phred quality score (Q-score) threshold of 8 and a minimum read length of 200 bp.

### Genome assembly and annotation

We removed lambda sequences from the Nanopore reads added in the library preparation step using NanoLyse version 1.1.0 (De Coster *et al.* 2018). We trimmed the Nanopore adapters from each read using Porechop version 0.2.4 (Wick *et al.* 2017). Quality filtering was achieved using NanoFilt version 2.3.0 (De Coster *et al.* 2018) with a quality score of **≥**5 and a minimum read length of **≥**1,000 base pairs. We searched the raw Illumina and Nanopore reads for species-specific primer sequences used for the molecular identification of *V. apis, V. ceranae*, and *V. bombi* before starting any analysis and only found evidence of *V. bombi*. Similarly, we aligned all of the quality-filtered Nanopore reads to the *B. terrestris* genome (GenBank accession ID: GCA_910591885.2) using BWA-mem version 0.7.17-r1188 (Li 2013) and removed all reads which matched bumblebee DNA (∼95% of the reads). As an additional filtering step, we screened a preliminary assembly using Canu version 2.2 (Koren *et al.* 2017) (the same steps as described next) through NCBI MegaBLAST (Morgulis *et al.* 2008) and filtered Nanopore reads that matched bacterial sequences using default settings.

All quality-filtered and non-bumblebee Nanopore reads were assembled using Canu version 2.2 (Koren *et al.* 2017) with parameters corOutCoverage = 100, corMhapSensitivity = high, minInputCoverage = 0, corMinCoverage = 0, and the precited genome size as 10 Mb, which is approximately the same length of the other two bee-infecting microsporidia—*V. apis* (8.5 Mb; Chen *et al.* 2013) and *V. ceranae* (7.9–8.8 Mb; Cornman *et al.* 2009; Huang *et al.* 2021).

We trimmed adapter sequences from the Illumina reads using Trimmomatic (Bolger *et al.* 2014) and aligned them to the draft Nanopore assembly using BWA-mem (Li 2013). We down sampled and normalized the combined aligned reads to ∼120× coverage using bbnorm from the BBTools suite version 39.01 (Bushnell 2014) and then iteratively polished the assembly three times using Pilon version 1.24 (Walker *et al.* 2014). We then ran through the assembly and polishing process again using these filtered reads and made final sequence adjustments according to the NCBI database requirements. We evaluated our assembly using the quality assessment tool QUAST v5.2.0 (Gurevich *et al.* 2013).

We annotated the genome using Braker2 version 2.1.6 (Brůna *et al.* 2021) with esmode (for gene prediction with genome sequence data only) and softmasking enabled which combines GeneMark-ET version 3.68 (Lukashin and Borodovsky 1998) and AUGUSTUS version 3.5.0 (Stanke *et al.* 2006). From the resulting annotation, we generated protein and coding sequence files using gffread (Pertea and Pertea 2020).

To assess annotation completeness, we searched the *V. bombi* VR1 predicted protein sequences and the predicted proteins from an additional 35 microsporidia genome assemblies (Supplementary Table 1) for universal Benchmarking Universal Single-Copy Orthologs (BUSCOs) version 4.1.2 (Manni *et al.* 2021) specific to the microsporidia (microsporidia_odb10). BLASTn (gene sequence) and tblastn (protein sequence) were used to search for *V. ceranae* BRL HAT1 against the *V. bombi* genome (E < 1e-5). tblastn was used to search for the 265 uniquely identified genes from orthology clustering against every other microsporidian genome.

Gene ontology mapping for *V. bombi* VR1 was achieved using Blast2Go(Conesa *et al.* 2005) and PFAM domains and KEGG classifications were assigned to each gene using InterPro (Hunter *et al.* 2009). We predicted the presence of signal peptides using SignalP4 (Petersen *et al.* 2011). tRNAs were predicted using tRNAscan-SE eufindtRNA version 1.1 (Lowe and Eddy 1997), and rRNAs using RNAmmer version 1.2 (Lagesen *et al.* 2007). We identified repeated elements in the assembly with RepeatMasker version 4.0.8 (Smit *et al.* 2013-2015) using a *de novo* repeat library generated with RepeatModeler version 1.0.11 (Flynn *et al.* 2020) with LTRStruct.

### Orthology prediction and phylogenetics

Orthologs were predicted between our new *V. bombi* VR1 annotation and the annotation for 36 microsporidia species (1 strain representative with the highest *N*_50_ and contiguity) available in MicrosporidiaDB (Aurrecoechea *et al.* 2011) and NCBI using the Synima pipeline (Farrer 2017) with OrthoMCL (Li *et al.* 2003) (Supplementary Table 1). Specifically, the Synima pipeline outputs Orthogroups, which can be divided into categories of interest, including single-copy orthologs, which we used to construct phylogenetic trees. We also separated out Orthogroups that had genes from every microsporidia species apart from *V. bombi* (those that we describe are uniquely lost in *V. bombi*) and those that are only found in a given species (those that we describe as unique to a given species). The script (Ortholog_dist_per_genome_summary.pl) used to separate out these categories of interest has now been added to the Synima Github repository.

We generated phylogenetic trees using 18 microsporidia single-copy orthologs. Briefly, Orthogroups were aligned using MUSCLE version 2.1.11 (Edgar 2004) and trimmed using trimAl version v1.4.rev15 (Capella-Gutiérrez *et al.* 2009) with the parameters -w 3 -gt 0.95 -st 0.01. We concatenated the alignments into a single FASTA using a bespoke script and converted that into nexus format. We used ProtTest version 3.4.2 (Darriba *et al.* 2011) to identify the best-fitting model for protein evolution (GAMMAILGF) and used that to generate a maximum likelihood phylogenetic tree using RAxML version 7.7.8 (Stamatakis 2014) with 1,000 bootstraps.

### Selection and enrichment analyses

*dN*/*dS* using the Yang and Nielsen method (Yang and Nielsen 2000) was calculated for each of the five conserved SWPs in *V. bombi* compared with *V. ceranae* using the Prank v.170427 (Löytynoja 2021) multiple alignment tool, and PAML version 4.10.6 Codeml (Yang 2007). GO-term enrichment analyses were conducted using the two-tailed Fisher's exact test with q-value FDR. Multiple testing corrections were achieved using the Storey–Tibshirani method (Storey and Tibshirani 2003) (requiring q values of <0.05). Hypergeometric tests were conducted using the R phyper function to test for enrichment of genes with secretion signals among orthogroups unique to a single species.

## Results

### 5.1 Genome assembly of *V. bombi* VR1

**Fig. 2.**
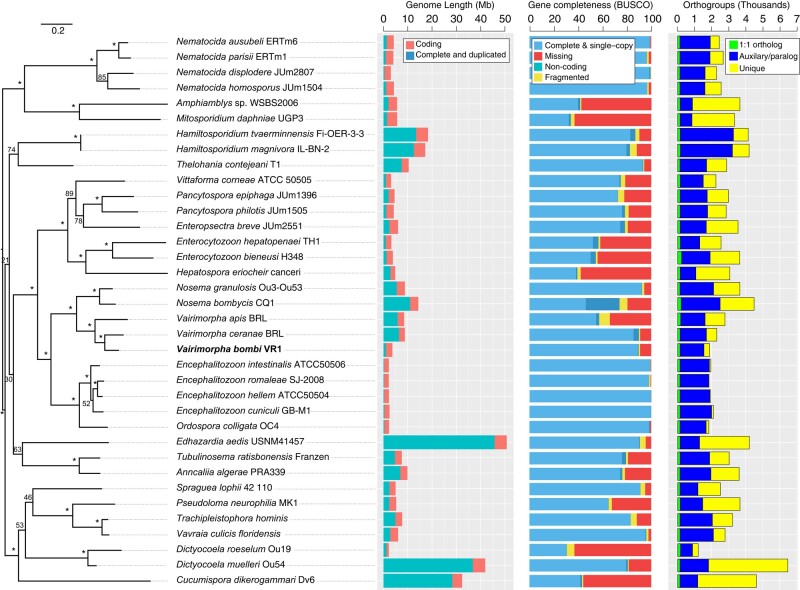
A maximum likelihood phylogenetic tree constructed using multiple alignment based on 18 single-copy core orthologs and RAxML (using the best-fitting model PROT + GAMMA + ILGF) with 1,000 bootstraps. To the right of the phylogenetic tree are three bar charts showing for each microsporidian genome assembly the genome length (separated into coding and non-coding), the BUSCO gene completeness score (separated into complete and single copy, complete and duplicated, fragmented and missing), and the thousands of predicted Orthogroups (separated into 1:1 core ortholog, auxiliary/paralogs and unique). * Indicates 100% bootstrap support, while numbers indicate <100% bootstrap support.

We sequenced and assembled the genome of *V. bombi* (isolate name VR1) using Oxford Nanopore and Illumina technologies. The genome assembly of *V. bombi* VR1 that we generated in this study comprises 57 contigs and has a total length of 4.73 Mb with an *N_50_* of 216 kb and with a low GC content (28.7%) (Table 1). From this genome assembly, we performed gene predictions, which identified 1,870 protein-coding genes and 179 tRNA genes. The protein-coding genes predicted encompass 2.23 Mb of sequence (∼61% of the genome), while repetitive content comprises only a small portion of the genome (44 kb; 1.2% of the genome). The remainder of the genome assembly is intergenic (985 kb; 27%) and intronic (449 kb; 12%). Gene completeness analysis using the BUSCO approach with the microsporidia_odb10 dataset revealed a completeness score of 90%. This BUSCO score is similar to its closest relative *V. ceranae* (also 90%) and with fewer duplicated complete BUSCOs (only 4 found in *V. bombi*; 27 found in *V. ceranae*). Compared with 35 additional microsporidia species, we found that the *V. bombi* genome assembly is the smallest of the *Vairimorpha* and *Nosema* genera, but larger than those found in the *Encephalitozoon* and *Ordospora* sister clades, and that our genome assembly quality is similar or better than those of its closest relatives (Fig. 2).

**Table 1.** *V. bombi* VR1 genome and annotation summary. Mb = Megabase, kb = kilobase, bp = basepair.

Assembly	
Total length (Mb)	4.73
Total length without N's (Mb)	3.66
GC content (%)	28.7
Contig N50 (kb)	216
Contig number	57
BUSCO completeness score (%)	96.8

Annotation	
Total length of repeats (bp)	43,850
Number of protein-coding genes	1,870
Total length of genes (Mb)	2.23
Mean gene length (bp)	1,189
Mean exon length (bp)	733
Mean intron length (bp)	386
Average exons per gene	1.6
BUSCO completeness score (%)	90

### Genome evolution, orthology, and phylogenetics of microsporidia

We identified 18 single-copy 1:1 orthologs that were conserved across all 36 species of microsporidia that we analyzed (Supplementary Tables 1 and 2). Phylogenetic analysis using these orthologs placed *V. ceranae* as the sister species to *V. bombi*, giving new insights into the relationships between these microsporidian parasites of bees. More broadly, our phylogenetic inference grouped the *Vairimorpha* species with the *Nosema* genus, which includes *N. granulosis*, a pathogen of the crustacean *Gammarus duebeni*, and *N. bombycis* which causes Pébrine or “pepper disease,” in silkworms. More distantly related is the *Encephalitozoon* genus that includes pathogens of diverse animal hosts including rabbits, humans, and grasshoppers. The Encephalitozoon clusters closely with *Ordospora colligate*, which is an intracellular parasite of the crustacean *Daphnia magna*.

As expected, the 18 highly conserved orthologs included several ribosomal proteins, eukaryotic translation initiation factor, protein kinases, kinesin, and perhaps less expectedly, the Histone acetyltransferase (HAT) *GCN5*. These orthologs were used to infer a maximum likelihood that supported the previous grouping of taxa including among and between *Vairimorpha*, *Nosema*, and *Encephalitozoon* (Fig. 2). Most of the genes across the 36 microsporidia fell within auxiliary/paralog clusters, encompassing duplicated genes, some of which are conserved across multiple species. In contrast to all other microsporidian genome assemblies analyzed, orthogroup clustering indicated that the *V. bombi* VR1 genome does not encode the HAT *ESA1* (e.g. *V. ceranae* gene = G9O61_00G005020). To explore this gene absence further, we performed a tblastn of the *V. ceranae* gene to the *V. bombi* genome, which found two significant hits, neither of which were HAT *ESA1*. The first hit was to contig 2 (positions 245,128–246,240; E = 1.52e-169), which overlaps the protein-coding gene g363 (HrpA-like helicase) that clustered with genes from 34 other species including two genes from *N. bombycis*. The second hit was to contig 7 (positions 24,960–25,568; E = 5.26e-24) that overlaps the protein-coding gene g130 (HAT *MST2*) that clustered with genes from 20 other species including two genes from *N. bombycis*. These analyses suggest that *ESA1* is absent in *V. bombi*, although other genes share sequence similarity with it.

Orthology clustering identified 265 unique genes in *V. bombi* VR1 (i.e. no orthology to other microsporidian species genes), which is fewer than the number of unique annotated genes in most other microsporidia genome assemblies. To validate and analyze these genes further, tblastn was used to search for sequence similarity in all 36 microsporidian genome assemblies (Supplementary Table 3). This analysis revealed that 158 genes (60%) had novel sequences and were not in any of the other sequenced microsporidian genome assemblies. A further 107 genes (40%) did have sequence similarity to ≥1 microsporidian genome assemblies, most of which (*n* = 96/107; 90%) also overlapped annotated protein-coding genes. These unique genes are therefore likely to be mostly novel sequences, along with some paralogs or genes with sequence similarity with other genes and some inaccuracies associated with genome sequencing, assembly, gene annotation, and orthology clustering.

All 265 unique genes were searched for functional enrichment compared with the rest of the protein-encoded genes (1:1 core orthologs and auxiliary), identifying only two high-level GO term enrichments among the unique genes (cellular process and intracellular anatomical structure). Surprisingly, 51/265 (19%) of *V. bombi*'s unique genes encoded a secretion signal, which was significantly enriched, given that only 95 genes were predicted to be secreted in total across all 1,775 genes (hypergeometric test upper tail *P*-value = 7.5E-22). Intriguingly, of the 36 microsporidian genome assemblies we compared, 26 also had a significant enrichment (*P* < 0.01) of secreted signals encoded by unique genes, ranging from 6% in *E. romaleae* to 71% in *E. aedis* (Supplementary Table 4).

### Dynamically evolving SWPs

We compared the repertoire of *V. bombi* VR1 genes to the SWPs and polar tube proteins of its closest relative *V. ceranae* (Huang *et al.* 2021) (Supplementary Table 5)*. V. bombi* is predicted to encode most (*n* = 5/7) of the SWP's described in *V. ceranae*. SWPs encoded by *V. bombi* include two genes that are unique to *V. ceranae and V. bombi* (*SWP1* and *2*), one gene that is unique to *V. ceranae, V. bombi*, and *V. apis* (*SWP3*), and two that are more deeply conserved among microsporidia (*SWP9* found in 10/36 species and *SWP12* found in 27/36 species). *dN*/*dS* (ω) for the five conserved SWPs in *V. bombi* VR1 compared with *V. ceranae* were between 0.06 and 0.25 suggesting that those genes are undergoing purifying selection (Table 2).

**Table 2.** *dN*, *dS*, and ω (*dN*/*dS*) for the five conserved spore wall proteins (SWPs) in *V. bombi* VR1 compared with *V. ceranae*.

Gene	dN	dS	ω
HSWP1	0.5355	3.0194	0.1774
HSWP2	0.4695	3.5485	0.1323
HSWP3	0.5715	2.3068	0.2477
HSWP9	0.3952	4.0004	0.0988
HSWP12	0.2216	3.506	0.0632

The two SWPs not encoded by *V. bombi* are *SWP4*, which is uniquely encoded by *V. ceranae*, and *SWP7*, which is more widely conserved (encoded by 27/36 species). Using the 5 *V. bombi* SWPs as a BLASTp database, we searched for additional SWPs that were not orthologous with *V. ceranae* genes, revealing four additional SWPs that all matched to *SWP2* with E-value < 1E-5 (IDs = g414, g1028, g1030, g1482). Together, these analyses suggest dynamically evolving spore walls (in terms of gene presence and absence) among *Vairimorpha* species, including both conserved and uniquely encoded SWPs.

## Discussion

We present the first genome assembly for *Vairimorpha bombi*, which is 4.73 Mb and has low GC content and low repetitive richness. This genome assembly is the smallest of the *Vairimorpha* and *Nosema* genera, but larger than those found in the *Encephalitozoon* and *Ordospora* sister clades. We identified 2,049 genes (including 1,870 protein-coding genes and 179 tRNA genes), which is lower than average for the microsporidia (x̄ = 3,054). Indeed, only five species had fewer predicted genes including *Dictyocoela roeselum* (1,201 genes), *Encephalitozoon romaleae* (1,883), *Ordospora colligata* (1879), *Encephalitozoon hellem* (2,006), and *Encephalitozoon intestinalis* (2,011). The impact and extent of gene reduction in the microsporidia is poorly understood. Understanding the genome evolution of the microsporidia is hampered by poorly resolved draft genome assemblies owing to relying on short sequence reads, and a challenge of separating host and pathogen DNA. Indeed, even with Oxford Nanopore reads, our assembly is still fragmented and includes ambiguous bases, despite its small size. Despite these challenges, our long and short read-based hybrid assembly achieved a 90% BUSCO gene completeness metric, which is higher than the average across all species (x̄ = 78%).

Our phylogenetic analysis based on 18 conserved single-copy orthologs and previous phylogenetic trees suggests that microsporidian evolution consists of both congruent and incongruent phylogenies with their host species, suggesting frequent host-jumps, especially among human-infecting microsporidia (Park and Poulin 2021). Whole genome or ortholog trees, such as the one we generated here, have the potential for providing greater resolution to microsporidian evolution, and also providing future insight regarding their overall placement in the fungal kingdom.

The HAT *GCN5* is a conserved ortholog across all 36 microsporidia analyzed. In other eukaryotes, *GCN5* is a subunit of the SAGA (Spt-Ada-Gcn5 acetyltransferase) transcriptional coactivator complex, which catalyzes acetylation of histone H3 and H2B N-terminal tails, with a corresponding association with gene activation. Given that neither H3 nor H2B were identified as microsporidian core orthologs, this raises the question of what the function of microsporidian *GCN5* might have. An intriguing hypothesis is that it functions as a host protein to disrupt or interfere with normal gene regulation, including innate and adaptive immune responses (Han, Gao, *et al.* 2020). Previously, it was suggested that Tra1p (a core component of the Ada and Spt proteins of the SAGA complex) is absent in microsporidia (Miranda-Saavedra *et al.* 2007). Additionally, the LisH domain that is part of Taf5 (a subunit of the transcription initiation factor TFIID) is absent in microsporidia (Jespersen *et al.* 2022), where it normally interacts with the Spt20 subunit of the SAGA complex (Miranda-Saavedra *et al.* 2007). Therefore, microsporidian encoded *GCN5* may function by itself, or apart from the SAGA complex. Our gene annotation and a BLASTn search of the *V. ceranae* BRL (closest relative) HAT1 gene to the *V. bombi* genome suggested that *V. bombi* has uniquely lost HAT *ESA1* compared with all the other microsporidia species. In other fungi, such as *Saccharomyces cerevisiae*, *ESA1* is essential for growth (Smith *et al.* 1998). It is unclear how or why *V. bombi* would dispense with this gene that is conserved across all other microsporidian and even *Vairimorpha* species. Further work should explore the functional roles that conserved microsporidian HATs have and the cellular locations of those functions.

We discovered a significant enrichment of secretion signals among genes that were predicted to be uniquely encoded by *V. bombi*, and indeed a significant enrichment of uniquely encoded genes that also encode a secretion signal in the majority of microsporidian species (n = 26/36; 72%). Secreted proteins have been shown to be important for infection, including, for example, the nematode-specific protein AAIM-1, whose loss of function confers resistance during *Nematocida parisii* infection. AAIM-1 is secreted into the intestinal lumen where it ensures spore orientation during intestinal cell invasion, as well as limiting bacterial colonization (Tamim El Jarkass *et al.* 2022). The function of all 2,818 total unique secreted proteins across the microsporidia that we identified is likely to be key for infection or competition and requires further study.

The SWPs and polar tube proteins are among the best-studied microsporidian virulence factors. Our genomic comparisons with *V. ceranae* suggested that most of the genes involved in the spore wall and polar tube are conserved (Huang *et al.* 2021). However, we also identified several SWPs that appear to be lost in many microsporidian species (e.g. *SWP9* is encoded and conserved in 10/36 species, while *SWP12* is found in 27/36 species). The two SWPs not encoded by *V. bombi* are *SWP4*, which is uniquely encoded by *V. ceranae*, and *SWP7* that is conserved more widely (encoded by 27/36 species). We identified four additional putative SWPs that had sequence similarity to *SWP2* (IDs = g414, g1028, g1030, g1482). Together, these analyses suggest a dynamically evolving spore wall among *Vairimorpha* species, including both conserved and uniquely encoded SWPs, which may be a signal of coevolution with their hosts.

Future work should aim to classify SWPs and genes that encode secretion signals according to their orthology, detailing how gene duplications or new genes have emerged in each genus or species. Additional genome sequencing, assembly, and annotation are also required, including efforts to improve current assemblies with additional long-read sequencing or short-read polishing to ensure repeat-content and gene annotation are accurately represented and fairly compared. Here, our sequencing, assembly, and comparative genomics of *V. bombi* have revealed unique genomic features of the pathogen, along with general features of genome evolution, both of which provide clues about the mechanisms underpinning microsporidian infection and host specificity.

## Supplementary Material

jkae029_Supplementary_Data

## Data Availability

The raw Illumina and Nanopore reads were submitted to NCBI GenBank (accession ID: SAMN33783333 and SAMN33864217). The annotated genome was submitted to NCBI (accession ID: PRJNA945438) and FigShare (10.6084/m9.figshare.24354313). Code used for genome assembly and analysis is provided on Github (https://github.com/VikiBlanchard/2022-23_Vairimorpha_bombi_de_novo_genome). Supplemental material available at G3 online.
