# Supplementary material for: Revealing the genome of the microsporidian Vairimorpha bombi, a potential driver of bumble bee declines in North America
Source: G3 (Bethesda). 2024 Feb 9;14(4):jkae029. doi: 10.1093/g3journal/jkae029 (PMC10989860; doi:10.1093/g3journal/jkae029)
Supplement: jkae029_Supplementary_Data [file jkae029_supplementary_data.zip › Supplemental_Material_Legends_G3-2024-404828.docx]

**Supplementary Information**

**Revealing the genome of the microsporidian *Vairimorpha bombi*, a potential driver of bumble bee declines in North America**

Victoria L. Webster^1^, Samuel Hemmings^2^, Marta Pérez^1^, Matthew C. Fisher^2^, Mark J. F. Brown^1,*^, Rhys A. Farrer^3,*^

**^1^**Department of Biological Sciences, Royal Holloway University of London, London, TW20 0EX, UK

^2^MRC Centre for Global Infectious Disease Analysis, Imperial College London, London, W2 1PG, UK

^3^MRC Centre for Medical Mycology, University of Exeter, Exeter, EX4 4QD, UK

*Corresponding authors

**Table S1.** Details of the 36 microsprodian genome assemblies including genus, species, isolate, data source, total length without ambiguity characters, N50, Nmax, Number of contigs (No.contigs), gene count, gene length total (nucleotides; nt), non-coding total (nt), Pubmed URL and NCBI accession ID.

**Table S2.** 18 single copy microsporidian orthologs found in each of the 36 species analysed in this study. For each protein sequence, BLASTp was used against the NCBI non-redundant database to determine the top most descriptive hit (not reporting some higher non-descriptive hits).

**Table S3**. tblastn used to identify sequence similarity between predicted unique genes from ortholog clustering and each of the 36 microsporidian genome assemblies. n = no hsp, y = hsp, and the gene locus ID is given in parathesis if the hsp overlapped a gene in the GFF.

**Table S4.** The number of genes, genes encoding a secretion signal (as predicted by SignalP4), unique genes based on orthology prediction, overlap (unique and genes that encode a secretion signal) as a count and as a percent of all genes that encode a secretion signal are provided. An enrichment test in R based on a hypergeometric test is given including the code used in R, the p-value, and whether enrichment was found (based on *p* < 0.01).

**Table S5.** Details of the genes encoding hypothetical spore wall proteins (HSWP) and polar tube genes, their functional domain, Genbank ID (as described in (Huang *et al.* 2021)) and their orthology to *V. bombi*. N/A = non-applicable (Genbank ID not identified in GFF3).
